# Numerical Investigation of Cell Encapsulation for Multiplexing Diagnostic Assays Using Novel Centrifugal Microfluidic Emulsification and Separation Platform

**DOI:** 10.3390/mi7020017

**Published:** 2016-01-25

**Authors:** Yong Ren, Wallace Woon Fong Leung

**Affiliations:** 1 Department of Mechanical, Materials & Manufacturing Engineering, The University of Nottingham Ningbo China, Ningbo 315100, China; 2 Department of Mechanical Engineering, The Hong Kong Polytechnic University, Hung Hom, Hong Kong, China; mmwleung@polyu.edu.hk

**Keywords:** centrifugal microfluidics, droplet separation, emulsification, cell encapsulation

## Abstract

In the present paper, we report a novel centrifugal microfluidic platform for emulsification and separation. Our design enables encapsulation and incubation of multiple types of cells by droplets, which can be generated at controlled high rotation speed modifying the transition between dripping-to-jetting regimes. The droplets can be separated from continuous phase using facile bifurcated junction design. A three dimensional (3D) model was established to investigate the formation and sedimentation of droplets using the centrifugal microfluidic platform by computational fluid dynamics (CFD). The simulation results were compared to the reported experiments in terms of droplet shape and size to validate the accuracy of the model. The influence of the grid resolution was investigated and quantified. The physics associated with droplet formation and sedimentation is governed by the Bond number and Rossby number, respectively. Our investigation provides insight into the design criteria that can be used to establish centrifugal microfluidic platforms tailored to potential applications, such as multiplexing diagnostic assays, due to the unique capabilities of the device in handling multiple types of cells and biosamples with high throughput. This work can inspire new development of cell encapsulation and separation applications by centrifugal microfluidic technology.

## 1. Introduction

Emulsions are produced in a mixture of two immiscible fluids, where the dispersed phase is suspended as droplets in the continuous phase. Emulsions are ubiquitous in our daily life and are widely used in the production of drugs, foods, cosmetics [1] and biochemical reactions [2,3]. They are good candidates to synthesize micro-objects, such as particles and capsules for various chemical, biomedical, and industrial applications [4,5], including particle-based display technologies [6], photonic materials [7,8], field-responsive rheological fluids [9], tissue engineering scaffolds [10], therapeutics [11], high performance composite filler materials [12], consumer and personal care products [13], ultrasound contrast agents [14], drug-delivery vehicles [15], and food additives [16]. In particular, cell studies by encapsulation, incubation, and manipulation in emulsion droplets are gaining popularity due to their numerous advantages [17,18]. Merging the two fields, cell biology and droplet technology, provide biologists with a micromanipulation method to handle a very small number of cells, and possibly even a single cell. When the droplet fluid is composed of cell culture media, continuous nourishment can be provided to the encapsulated cells. The droplets are normally produced either by mechanical agitation or intense shear. These conventional methods usually result in formation of droplets with a broad size distribution. The low energy efficiency and poor control over payload also severely hinder their practical applications [19]. Alternatively, droplets can be formed by microfluidic technology, which refers to the science and technology of miniaturization processes that manipulate minute amount of fluids, such as cell suspensions, protein, antibody solutions, blood samples, or buffers using devices with characteristic dimensions of tens to hundreds of micrometers [20]. Microfluidics provides a more precise control over emulsion formation, because the size, shape, and concentration of droplet can be finely tuned [21], leading to formation of droplets with high monodispersity and uniformity of desired properties. Therefore, such droplets can act as a template for preparation of highly monodispersed particles or capsules, which are especially advantageous for cell studies. The monodispersity enables quantitative control of solute concentrations, while encapsulation in a droplet provides an isolated compartment for the cell in a compatible environment. The high throughput enabled by microfluidics due to ease of parallelization allows the processing and analysis of tens of thousands to millions of cells that are required to be analyzed to find rare cell types of interest for screening applications. The extremely low volumes of the droplets also render large-scale screening cost effective. Droplets can be passively formed by fluid instabilities using four of the most prevalent microfluidic systems, *i.e.*, coaxial [22], flow-focusing [23], T-junction [24] and step emulsification [25,26]. The dynamics of the droplet formation process can be characterized into a dripping and a jetting regime. The dripping-to-jetting transition can be estimated using the Capillary number of the continuous phase, *Ca_CP_*, *i.e.*, the ratio of viscous force to interfacial tension, and the Weber number of the dispersed phase, *We_DP_*, *i.e.*, the ratio of inertial force to interfacial tension [27].

(1) $$Ca_{CP}=\frac{\eta_{CP}V_{CP}}{\sigma }$$

(2) $$We_{DP}=\frac{\rho_{DP}L{V_{DP}}^{2}}{\sigma }$$

where $V_{CP}$ and $V_{DP}$ are the velocities of continuous and dispersed phase, respectively, σ is the interfacial tension between two phases, $\eta_{CP}$ is the apparent viscosity of the continuous phase, $\rho_{DP}$ is the density of dispersed phase, and *L* is the characteristic dimension of the microsystem, *i.e.*, the hydraulic diameter, *D_h_*, of the microchannel. Droplet formation occurs in the dripping process, when both *Ca_CP_*, and *We_DP_* are small, as interfacial tension dominates. Jetting occurs when *Ca_CP_*, and *We_DP_* are large, as the viscous stress or the inertial force on the droplet becomes sufficiently large overcoming interfacial tension. Droplets are generated after breakup of a jet, typically at some distance downstream of the nozzle.

Droplets can also be generated using a centrifugal microfluidic platform, which relies on intrinsic pseudo-forces including centrifugal force, Coriolis force and Euler force for fluid transport and processing, such as mixing [28,29,30,31,32]. Centrifugal microfluidics has evolved into a mature technology and widely used in various fields such as clinical chemistry, immunodiagnostics and protein analysis, cell handling, molecular diagnostics, as well as food, water, and soil analysis [33,34,35]. Centrifugal microfluidics has received great interest due to its advantages such as high parallelization, free of external actuation and compatibility with mass production. Moreover, centrifugal pumping is relatively independent of the physicochemical properties of the fluid, such as pH or ionic strength, therefore a wide variety of fluids including aqueous solutions, solvents, surfactants, and biological fluids (*i.e.*, cell culture medium, blood, urine, and mucus) can be used. Provided the rotating speed is sufficiently large and adjustable, the aforementioned fluids can be pumped successfully using the centrifugal microfluidic platform, overcoming the viscous and surface tension forces [34,35]. A typical centrifugal microfluidic platform is composed of a microfluidic compact disc device, hardware and software drives for the control of the rotation platform, and a system for data acquisition. The platform size and rotation speed depends on the integration and complexity level of the three components and the specifications of control motors. With a compact motor and a high degree of integration, the entire platform size can be comparable to that of a portable compact disc player [34]. Our group has developed a rotating platform where the device is fixed in an aluminum holder, which can accommodate a compact disc with size up to 150 mm, and the platform rotates with maximum speed of 1800 rpm [35]. Chakraborty *et al.* and Haeberle *et al.* have demonstrated for the first time the production and manipulation of gas-in-oil, and water-in-oil droplets, respectively, using the centrifugal microfluidic technique [36,37]. Compared to conventional microfluidic emulsification techniques, the use of syringe pumps and tubing is obviated in centrifugal microfluidic emulsification [38,39,40]. Hydrodynamic focusing is one of the most popular designs, where the dispersed phase is purged from a nozzle and contacts with the continuous phase from both sides at the junction of a rotating flow-focusing structure. The main governing parameters include, the three incoming flow rates, the angle at which the side flows impact the central stream and the radial position of the intersection. The droplet generation is characterized by, respectively, the droplet diameter, the droplet spacing, and the droplet rate. The impact of the centrifugal acceleration on the droplet formation can be quantified by the Bond number, *Bo*, which represents the ratio of centrifugal over interfacial effects on the droplet [37]:

(3) $$Bo=\frac{\rho_{d}r\Omega^{2}L^{2}}{\sigma }$$

where $\rho_{d}$ refers to the density difference between the dispersed and continuous phases, *r* denotes the radius from the center of rotation, and Ω represents rotation speed. The dimension of the issuing nozzle is normally used as the value of characteristic length *L*. The flow regime can vary between monodispersed emulsions and segmented-flow. The droplet size and generation rate can be adjusted via control over the Bond number by changing the channel geometry and the rotation speed.

A high throughput centrifugal step emulsification disk has been developed to enable the generation of emulsions with high internal volume fraction [41,42], which can be applied in the production of porous functional polymers [43]. Droplets formed by centrifugal microfluidics can be applied as a template to synthesize functional materials, such as alginate microbeads [44], chitosan microbeads [45], and 3D multi-compartmental particles [46]. The advantages include artificial gravity-based pulse-free propulsion, and the capability to form well-defined highly parallel microdroplets with minimal dead volume. For example, centrifugal step emulsification can be employed for absolute quantification of nucleic acids by digital droplet RPA (recombinase polymerase amplification) [42]. Furthermore, the integration of droplet-based operations, together with complex sample preparations such as nucleic acid purification can enable sample-to-answer implementations of digital assays. Haeberle *et al.* presented an approach for centrifugally induced fabrication of alginate microbeads using polymer micro-nozzles. The collected alginate droplets underwent a hardening process to form calcium ion-hardened alginate beads. The bead diameter can be adjusted according to the nozzle geometry and the rotational speed. HN25 and PC12 cells were encapsulated in microbeads for cell studies [47].

Despite these aforementioned advances, some limitations exist and cannot be overlooked. The droplets are formed in the radially aligned channel in all the aforementioned literature using flow-focusing structure. The rotation-induced centrifugal force purges the dispersed phase from the nozzle. A normal approach to enhance production rate of droplets by centrifugal microfluidic technology is to increase rotation speed, however, when the speed is higher than a threshold value, *i.e.*, the Bond number is larger than the critical value, the flow regime will undergo transition from dripping to jetting, where axially extended threads of the dispersed liquid are injected into the continuous phase. These jets are unstable and tend to break up into droplets upon transient fluctuations within the jet due to Rayleigh instability. A broad droplet-size distribution is observed arising from breakup behavior of the jet [37]. The production rate of droplets will also be significantly lowered. This hinders the application of centrifugal emulsification. Another drawback of existing technology is that only a limited type of cells can be encapsulated using the same rotation platform.

Here, we present a novel centrifugal microfluidic device featured with T-junctions to form droplets in circumferentially oriented microchannels. Throughput of droplets with this novel device can be ensured at high rotation speed. Simultaneous encapsulation of multiple different types of cells can also be enabled by such a novel system, as different types of cells can be accommodated in respective reservoirs before they are purged into a main channel when the device undergoes rotation. This allows important applications for multiplexing diagnostic assays. Processing different immiscible liquids in multiphase flows also facilitates the simultaneous processing of fluids with different rheological properties in the same system. The separation process constitutes an important unit operation within many emulsification applications. The present centrifugal microfluidic platform also enables droplet separation in a facile way, based on the balance between Coriolis and centrifugal forces. In this study, the formation of single emulsion droplets at upstream T-junction and sedimentation of droplets at downstream separation junction have been investigated through numerical simulation. This work can inspire new cell encapsulation applications using centrifugal microfluidic platform.

## 2. Design of the Centrifugal Microfluidic Device

**Figure 1:**
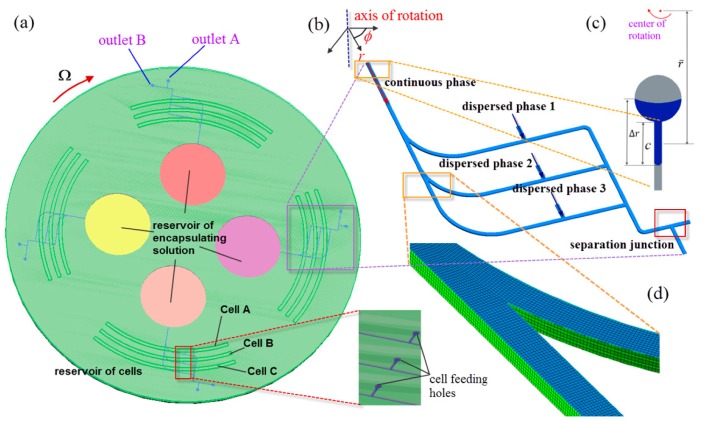
(**a**) Schematic of the centrifugal microfluidic device; (**b**) schematic of the computational domain of the multiphase centrifugal microfluidic microchannels; (**c**) close-up view of thread of continuous phase as indicated in blue progressing along microchannel after being pumped from the reservoir; and (**d**) close-up view of generated mesh grids of the bifurcated junction.

The whole system comprises one top compact disk featured with truncated pie-shape chambers as reservoirs to store different types of cells containing a culture medium as dispersed phases; and one bottom disk featured with four similar operating units, each of which is composed of one circular chamber storing the encapsulating solution as a continuous phase, which will be purged into a radially-oriented microchannel once the rotation speed of the disk is beyond the threshold value for which the centrifugal force overcomes the flow resistance along the channel, as depicted in Figure 1a–c. The disk has a diameter of 108 mm and height of 3 mm. The depth of all the chambers is 2 mm. The width *W* and height *H* of the microchannel are 200 and 100 µm, respectively. The channel is further split up at three downstream bifurcated junctions. After passing the bifurcated junction, the encapsulating solution flows along the various sub-channels, which are circumferentially patterned. Concurrently, the cell medium is transferred from cell reservoirs to the microchannels via the cell feeding holes, as shown in the inset of Figure 1a. The dispersed phase of the cell medium meets the continuous phase of the encapsulating solution at the T-junction, where droplets are formed to encapsulate different types of cells, and the cell-encapsulated droplets can be collected at downstream for further processing and analysis.

## 3. Numerical Model

A 3D numerical model of the rotating multiphase microchannel where droplets are generated was established as shown in Figure 1b. The geometry of cell feed holes and reservoirs was ignored to save computational cost. The domain is stationary initially, and subsequently is subject to a prescribed rotation scheme. The governing equations for incompressible two-phase fluids on a rotating reference frame can be described by [48,49]:

(4) $$\frac{\partial \alpha_{CP}}{\partial t}+V\cdot \nabla \alpha_{CP}=0$$

(5) $$\frac{\partial \rho }{\partial t}+\nabla \cdot \left(\rho V\right)=0$$

(6) $$\frac{\partial }{\partial t}\left(\rho V\right)+\nabla \cdot \left(\rho VV\right)=-\nabla P+\nabla \cdot \left[\eta \left(\nabla V+\nabla V^{T}\right)\right]+\frac{\rho \sigma \nabla \alpha_{CP}}{\frac{1}{2}\left(\rho_{DP}+\rho_{CP}\right)}\nabla \cdot \frac{\nabla \alpha_{CP}}{\left|\nabla \alpha_{CP}\right|}+\rho S$$

(7) $$S=-\Omega \times \left(\Omega \times r\right)-2\Omega \times V+r\times \frac{d\Omega }{dt}$$

(8) $$\rho =\alpha_{CP}\rho_{CP}+\left(1-\alpha_{CP}\right)\rho_{DP}$$

(9) $$\eta =\alpha_{CP}\eta_{CP}+\left(1-\alpha_{CP}\right)\eta_{DP}$$

where *V* is the flow velocity, *P* is the pressure, ρ is the volume averaged density, $\alpha_{CP}$ and $\rho_{CP}$ are the volume fraction and density of the continuous phase respectively, $\eta_{DP}\;$ is the dynamic viscosities of the dispersed phases, and *r* denotes the radial vector from the center of rotation. The source term due to rotation is composed of three terms, *i.e.*, centrifugal acceleration, Coriolis acceleration and the Euler term, respectively, as shown in Equation (7). The gravity effect was neglected for the sake of simplicity. The two-phase flow problem was solved using the VOF (Volume of Fluid) method by CFD software Ansys Fluent 15.0 (Canonsburg, PA, USA). The governing equations were discretized to algebraic equations using a control-volume-based technique. The PISO (Pressure-Implicit with Splitting of Operators), algorithm was used in the transient calculations for droplet generation and deformation. The technique of PLIC (Piecewise-Linear Interface Construction), was adopted in the numerical investigation, as it can ensure the accuracy of the interfacial tension calculations [50]. An iterative solver was deployed to solve the control-volume discretized equations. The iterative time step is 10^−7^ s and the solution converges when the residual is below a tolerance set as 1.0 × 10^−6^. No-slip condition was applied at the solid boundaries of the walls of the microchannels. Zero gauge pressure was applied at the outlet of the channelss. The flow was assumed laminar and flow velocity was specified at the inlet of the domain. The average velocity, *U*, of centrifugally-pumped liquid in a channel depends on, the rotational speed Ω, radial location of the fluid reservoirs, channel geometry, and fluidic properties (*i.e.*, dynamic viscosity η, density ρ, *etc.*) and can be derived as [51]:

(10) $$U=\frac{{D_{h}}^{2}{\rho \Omega }^{2}\bar{r}\Delta r}{32\eta c}$$

where $\bar{r}$ is the average distance of the liquid in the channels from the center of the disc, Δr is the radial extent of the liquid, and *c* is the length of the liquid in the channel (see Figure 1c).The simulations were performed using numerical grids composed of hexahedron elements generated by ANSYS package ICEM CFD 15.0 (see Figure 1d).The numerical data were subsequently analyzed by the Ansys CFX-Post Processor 15.0 after simulation was completed. Biopolymer alginate is the most commonly used material to encapsulate cells because of its good biocompatibility [52]. Alginate is produced from brown algae and is referred to as sodium-alginate in the liquid state. As soon as sodium-alginate comes in contact to a solution containing calcium ions, it rapidly hardens to form calcium-alginate via sol-gel transition. Therefore, cells dispersed in sodium-alginate solution and calcium chloride solution are normally used as dispersed and continuous phases, respectively, for practical cell encapsulation applications. In the present numerical study, as demonstration of cell encapsulation, silicon oil was selected as the continuous phase, while water was selected as the dispersed phase. The physical properties of the fluids used in the investigation are shown in Table 1. Wetting property is one of the key parameters influencing multiphase flow phenomena, and it is primarily determined by static contact angle θ obtained at the intersection of a solid substrate and a fluid/fluid interface at rest [53]. The fluid can be classified as partially wetting when θ < 90° and partially non-wetting fluid when θ > 90°. The case where fluid adopts an angle θ = 0° is referred to as complete wetting and leads to the formation of a metastable thin film covering the solid substrate, while the case associated with θ = 180° is referred to as complete non-wetting. It is assumed that our device is made of poly(dimethyl) siloxane (PDMS) as it is one of the preferred materials in microfluidic biomedical applications because of good biocompatibility, high gas permeability, chemically inert surface, and optical transparency. The native PDMS surface has a water contact angle of 112° ± 1° [54] and silicon oil contact angle of 26.44° ± 1.71° [55]; hence, these contact angles have been adopted in the second part of our numerical simulation.

**Table 1:** Physical property of the test fluids in droplet formation using our centrifugal microfluidic platform.

Test Fluids	η (cP)	ρ (kg/m^3^)	σ (mN/m) (Silicon Oil and Water)
Silicon oil	64.3	908.7	14.26
Water	1.003	997	

## 4. Results and Discussion

### 4.1. Validation of Numerical Model

**Figure 2:**
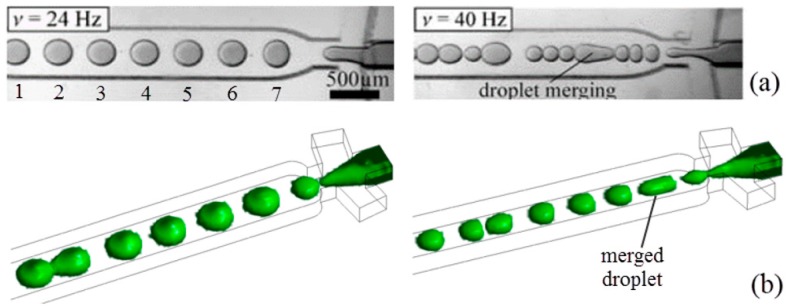
Comparison of results at rotation frequency of 24 Hz (*Bo* = 0.09) and 40 Hz (*Bo* = 0.25) between (**a**) experiments from Haeberle *et al.* [37] (reprinted with permission from Springer) and (**b**) our simulation using rotating flow-focusing two-phase microsystem where water is used as dispersed phase and silicone oil as continuous phase, *Ca_CP_* = 0.16.

The accuracy of our numerical model was verified by comparing it with the results of the experiment performed by Haeberle *et al.* [37]. Our study adopted fluids with physical properties (*i.e.*, density, viscosity, interfacial tension) the same as those used in the experiment, as listed in Table 2. Sunflower oil with viscosity of 62.2 cP and density of 909 kg/m^3^ was used as the continuous phase. Water with viscosity 1.09 cP and density of 1005 kg/m^3^ was used as the dispersed phase. The interfacial tension is 28.33 mN/m. Contact angle of water and sunflower oil are 82.7° and 10.3°, respectively, in contact with the surface of the microchannel fabricated from cyclo-olefin copolymer. The same channel geometry was used in simulation, *i.e.*, the droplet-channel length is 25 mm and height is 200 µm. The widths of the droplet-channel and downstream constriction channel are 660 and 376 µm, respectively. The junction angle is 80°. Two rotation frequencies have been attempted in our numerical study: 24 and 40 Hz, respectively, corresponding to *Bo* = 0.09 and 0.25, respectively. Patterns of droplet formation obtained from the experiment by Haeberle *et al.* [37] and our numerical simulation are shown in Figure 2. Ansys CFX Post Processor 15.0 was used to generate the 3D view of water-in-oil droplets obtained from Ansys Fluent 15.0, as shown in Figure 2b, where the green color illustrates the water/oil interface with zero thickness, *i.e.*, sharp interface. In reality, diffusion provides a finite interface, which has been neglected for simplicity. Since the experimental images are shown in 2D, top view images of 3D droplets from simulation are analyzed using image-processing software ImageJ, and the droplet area is automatically determined by changing the picture contrast and searching for the outline of the droplet. Quantitative comparison of the area for each droplet is listed in Table 3, where numbers 1–7 refer to the seven droplets generated in Figure 2a at a rotation frequency of 24 Hz. Average, standard deviation, and coefficient of variation (CV) of droplet area were calculated based on the measurement. The relative difference between simulation and experiments is less than 6%. In addition, the areas of droplets generated from our simulation at a rotation frequency of 40 Hz were also compared with those from the experiments. The deviation is within 5% for average droplet areas. In particular, the area of the simulated merged droplet is in agreement with experiment by a deviation lower than 3%. Compared to the experimental geometry, the upstream reservoirs and downstream constriction channel were ignored in our numerical model to simplify the calculation, which may contribute largely to such deviations. The large deviation for some of droplets can also be attributed to the occurrence of merging in the simulation, such as that taking place between the first and second droplets. In measurement of the size of droplets from the figure taken in the experiments by Haeberle *et al.*, errors may be incurred due to photographic artifacts such as shadows cast by the channel walls and reflections, which may affect the quantitative analysis of droplet interface profiles. Both experiments and simulations reveal that as the Bond number increases, *i.e.*, by increasing rotation speed, the droplet generation rate also increases while their size decreases.

**Table 2:** Physical property of the fluids used in our numerical simulation as benchmarked with the experiments reported by Haeberle *et al.* [37].

Test Fluids	η (cP)	ρ (kg/m^3^)	σ (mN/m) (Sunflower Oil and Water)
Sunflower oil	62.2	909	28.33
Water	1.09	1005	

**Table 3:** Comparison of average size of each droplet between simulation and experimental results (Numbers 1–7 refer to the seven droplets generated in Figure 2a).

Droplet Area (mm^2^)	#1	#2	#3	#4	#5	#6	#7	Average	Standard Deviation	CV
Experiment	0.078	0.081	0.078	0.086	0.078	0.085	0.076	0.080	0.004	0.046
Simulation	0.077	0.078	0.077	0.081	0.080	0.085	0.072	0.079	0.004	0.048
Relative difference	1.14%	4.03%	0.89%	5.44%	3.05%	0.54%	5.26%	2.07%	1.35%	3.49%

**Figure 3:**
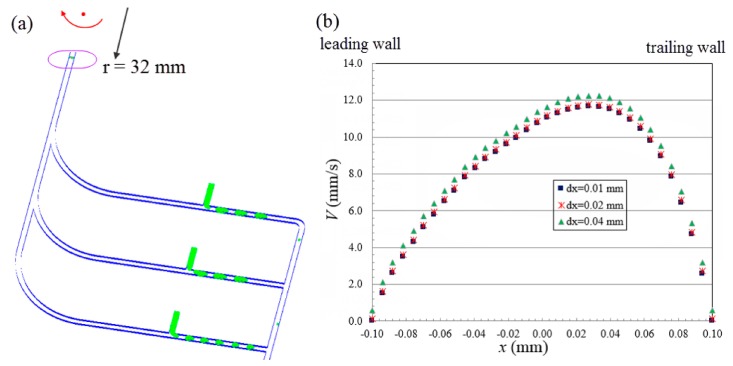
(**a**) Generation of water-in-oil droplets in the centrifugal microfluidic system when Ω = 20 rad/s. (**b**) Throughflow velocity profile across the center line of microchannel along transversal direction with different size of mesh grids at downstream located 32 mm from the center of rotation.

Subsequently, the same numerical settings were adopted in the second part of our computation work with exceptions that we are (a) using our new novel compact disc device (see Figure 1b); and (b) switching from sunflower oil to silicon oil (see Table 1). Sunflower oil and silicon oil have almost the same viscosity and density, except that the former possesses higher oil/water interfacial tension of 28.33 mN/m, while the latter has lower oil/water interfacial tension of 14.26 mN/m, which is relatively unfavorable in generating droplets. Silicon oil was chosen not only because it is more widely employed in biomedical applications, but also this allows us to demonstrate the capability of our novel device in generating droplets for oils with lower interfacial tension. In the simulation, the dispersed phase is purged out of the T-junction and moves downstream along the main channel. As the shear force from continuous phase overcomes the interfacial tension of dispersed phase, the latter adopts a thread shape which continuously becomes thinner and eventually breaks up into droplets moving downstream. The generated 3D droplets are illustrated by the green color, as shown in Figure 3a. In the rotating reference frame of the channel, the centrifugal force, *F_cen_*, acting on the fluid drives the throughflow towards the outlet with a velocity of *V*(*x*, *y*), where *x* and *y* represent the coordinates along the width-wise and height-wise direction, respectively. The throughflow along the rotating channel induces a Coriolis force, *F_cor_*, which is directed normal to the throughflow, yet opposite to the rotation direction. The magnitude of the Coriolis force depends on the magnitude of throughflow, which is a parabolic profile with maximum velocity at the center of the channel and zero velocity at the channel walls due to no slip. The Coriolis force produces a crossflow at the mid-plane (*y* = 0) of the channel directing towards the trailing wall (*x* = *W*/2) with flow returning near the front wall (*y* = *H*/2) and back wall (*y* = −*H*/2) of the channel towards the leading wall (*x* = −*W*/2). The crossflow continuously evolves as the fluid moves downstream. However, due to the Coriolis effect acting in the width-wise direction, the peak of the developing throughflow across the width of channel shifts towards the trailing wall (*x* = *W*/2) and results in an asymmetric parabolic profile, as shown in Figure 3b. A mesh size sensitivity study was conducted to determine the optimal size of mesh grids. Given the velocity profiles corresponding to the two mesh sizes *dx* = 0.02 (coarser mesh) and 0.01 mm (finer mesh), respectively, are in agreement, the mesh size of 0.02 mm was adopted in the following investigation to save computational cost.

### 4.2. Encapsulation of Multiple Types of Cells

**Figure 4:**
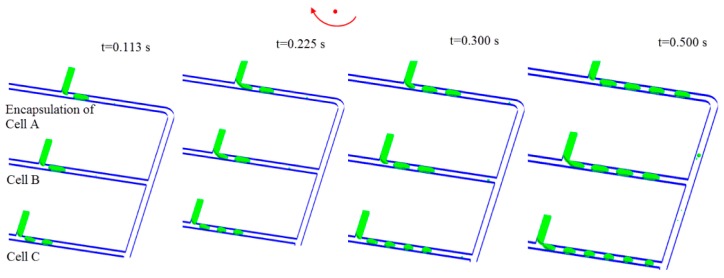
Simulation of encapsulation of multiple types of cells using same centrifugal microfluidic platform. The arrow denotes the direction of rotation. Ω = 50 rad/s.

The capability to encapsulate simultaneously multiple types of cells is demonstrated in Figure 4, where time lapse images of droplet generation are shown when the microfluidic platform rotates with Ω = 50 rad/s. Three types of cells, *i.e.*, cell A, B, and C, can be encapsulated by the droplets migrating along three sub-channels, respectively. Each droplet works as an individual self-contained micro-environment for the incubation and manipulation of cells. Separate and independent control over droplet size and production rate can be enabled by this design. The droplets are collected downstream for multiplexing cell studies and biochemical assays. Furthermore, the droplets containing different types of cells can be merged downstream to form an isolated compartment for incubation of multiple types of cells.

**Figure 5:**
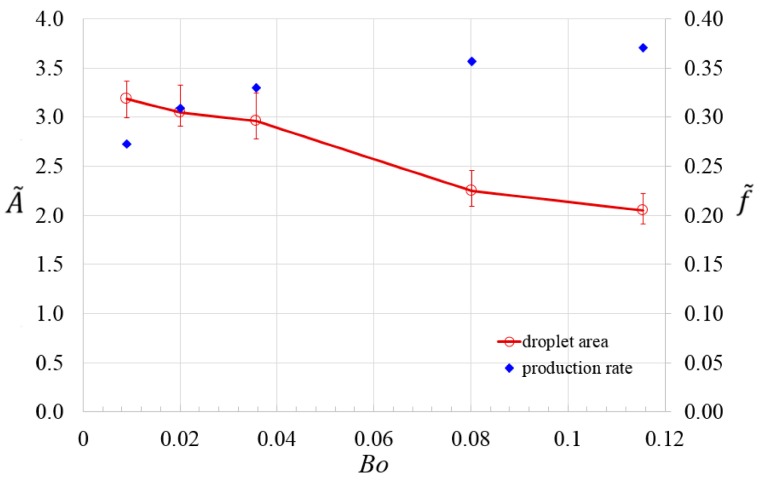
Dimensionless averaged droplet area and dimensionless production rate at different Bond numbers by controlling the rotation speed.

The cell encapsulation capability by the droplets is characterized by monitoring the droplet area *A*, and generation rate *f*, at different Bond numbers, and this is achieved by adjusting the rotation speed of the device. The number of produced droplets from two subsequent images of simulation is evaluated, and the droplet generation rate can be derived by calculating the change of number of droplets formed during the time interval. The average value of droplet area, *A_average_*, is obtained by taking average of the areas of individual droplets generated in circumferentially aligned channels at a given Bond number. The averaged droplet area has been measured at three different times after the numerical iteration converges to determine the mean value and error bars. The averaged droplet area and generation rate are subsequently non-dimensionalized by the cross-sectional area of microchannel and average shear rate, $\dot{\gamma }$, in the microchannel, respectively,

(11) $$\tilde{A}=\frac{A_{average}}{WH}$$

(12) $$\tilde{f}=\frac{f}{\dot{\gamma }}$$

where $\tilde{A}$ and $\tilde{f}$ refers to dimensionless averaged droplet area, and dimensionless generation rate, respectively. At a higher flow velocity in the channel due to a higher rotation speed, the droplets also experience higher shear stress and they are being generated faster by and from the T-junction. This leads to an increase in the droplet generation rate and a decrease in averaged droplet area, as shown in Figure 5. The throughput with the present novel design can be enhanced by operating the three circumferentially aligned channels simultaneously.

**Figure 6:**
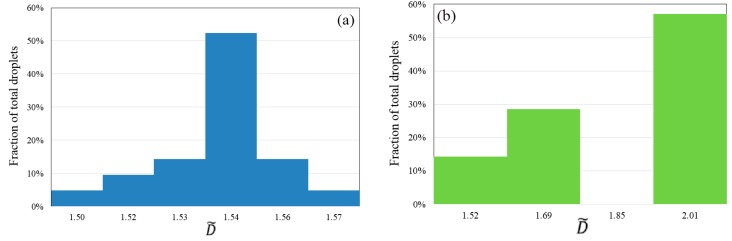
(**a**) Histogram of size distribution of 22 droplets generated in three circumferentially aligned channels before occurrence of merging and (**b**) histogram of size distribution of 7 droplets formed from merging, when Ω = 150 rad/s and *Bo* = 0.08. The droplet size is non-dimensionalized by hydraulic diameter of microchannel.

The histogram of size distribution of droplets formed in pre-merging and post-merging regime is shown in Figure 6a,b, respectively. The size of the droplets, *D*, is evaluated by assuming that the droplets adopt a spherical shape, and is non-dimensionalized by the hydraulic diameter of the microchannel,

(13) $$\tilde{D}=\frac{D}{D_{h}}$$

where $\tilde{D}$ denotes the dimensionless droplet size. There are 22 droplets with dimensionless average size of 1.54 that are generated from three circumferentially aligned channels prior to the occurrence of merging when *Bo* = 0.08, as shown in Figure 6a. A small coefficient of variance (CV) = 2%, defined by the ratio of standard deviation of droplet size to average droplet size, indicates the high reproducibility of the droplet breakup process in the pre-merging regime using the presented novel centrifugal microfluidic emulsification system. Merging occurs downstream of the microdevice, leading to the formation of larger droplets. However, not every droplet is merged with another because merging only occurs when two droplets approach each other downstream at a synchronized moving velocity. Therefore, one may find two peaks of droplet size, the larger one represents the size of merged droplet, while the smaller one represents the size of an unmerged droplet, as shown in Figure 6b. The microfluidic platform presented herein provides precise control over the size and production rate of droplets by adjusting the Bond number. It can be envisioned that the generated monodispersed droplets with controlled sizes can be applied to deliver an accurate dosing of contained payloads, such as drug, flavoring, or chemical reactants. Moreover, the ability to merge droplets into one droplet leads to drugs with different dosing capability for concurrent batch bioassays on droplets.

### 4.3. Droplet Sedimentation

**Figure 7:**
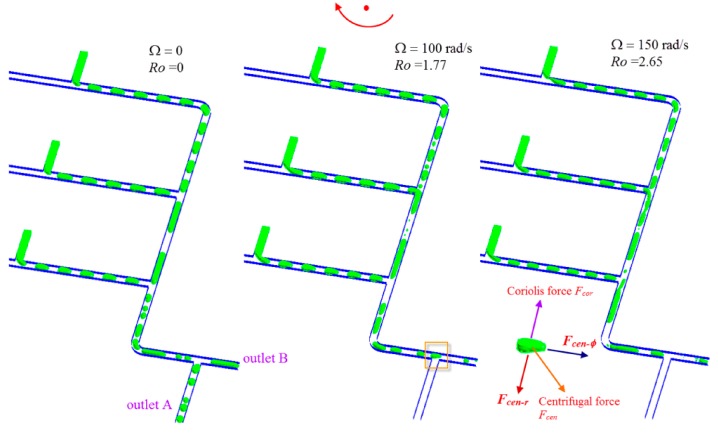
Sedimentation of cell-encapsulated droplets at the separation junction.

Droplet sedimentation is one of the critical applications in multiphase systems. The ability to control selectively the different phases is shown in Figure 7, using a bifurcated flow-splitting structure (see separation junction in Figure 1b). When the channel is stationary, *i.e*., Ω = 0, the multiphase water-in-oil emulsion flow is split into two outlet channels due to the nature of pressure-driven flow. In contrast, when the whole system is under rotation, for example, with rotation speed of 100, and 150 rad/s, respectively, the centrifugal force and Coriolis force both act simultaneously on the cell encapsulated droplet, see inset of Figure 7. The centrifugal force can be further resolved into two components, *F_cen-_*_φ_ and *F_cen-r_*. The former will drive the droplet to move along the circumferential direction, while the latter is pointing radially outwards, pushing the droplet to move towards outlet A. Meanwhile, the Coriolis force is generated which is perpendicular to the direction of *F_cen-_*_φ_, while opposite to the direction of *F_cen-r_*, it attempts to force the droplet against the left wall of the channel along the droplet motion. Consequently, the droplets leave the disk via the outlet B instead of outlet A. As such the governing parameter should be the ratio of the Coriolis force to the centrifugal force, which is the Rossby number, *Ro*,

(14) $$Ro=\frac{2\Omega U}{\Omega^{2}r}\sim \frac{U}{\Omega r}$$

It can be shown from Equation (10) that the average velocity along the channel under centrifugal acceleration is:

(15) $$U\sim \frac{\rho \Omega^{2}r\Delta r{D_{h}}^{2}}{\eta c}$$

Assuming Δr=c, therefore:

(16) $$Ro=\frac{2\Omega U}{\Omega^{2}r}\sim \frac{U}{\Omega r}\sim \frac{\left(\frac{\rho \Omega^{2}r{D_{h}}^{2}}{\eta }\right)}{\Omega r}\sim \frac{\rho \Omega {D_{h}}^{2}}{\eta }$$

*Ro* is independent of radius and depends primarily on, the rotation speed, properties of dispersed phase, and the hydraulic diameter of microchannel $D_{h}$. The separation of droplets from the continuous phase is governed by the Rossby number, nevertheless, interfacial tension does not come into play in the separation. Hence, when the multiphase system is under rotation as *Ro* < 1 the flow leaves outlet A, *vice versa* as *Ro* >> 1 the flow leaves outlet B. The sedimentation of a cell-encapsulated droplet controlled by the ratio of the centrifugal to Coriolis forces is a new manipulation parameter in the present centrifugal multiphase microfluidic platform for multiplexing diagnostic assays.

## 5. Conclusions

A novel centrifugal microfluidic device has been developed for droplet generation, and encapsulation of multiple types of cells. The device enables separate control over droplet size and production rate through adjustment of the rotation speed. It also allows droplet sedimentation in a facile way. Each droplet works as an individual isolated micro-environment for cell incubation. Multiple reservoirs, each with different types of cells for identifying specific bio-samples, can all be installed on a single rotating disc which undergoes rotation to realize effective cell growth followed by bio-sample detection downstream in a cost-effective and energy-efficient manner. Given the operation units can be all integrated on a small rotating platform, which is similar to a compact disc player, multiplexing diagnostics can be automated for point-of-care applications. The design also facilitates parallelization so that large-scale cell studies, such as cell screening can be realized. Besides the capability to process multiple types of cells and achieve droplet sedimentation effectively, another advantage of the centrifugal micro-encapsulation technology includes ease of implementation and low cost, as no additional ancillary pumps and equipment other than the disposable unit is required, thus obviating numerous cleaning and sterilization steps which are common expensive practices with existing technologies. The aforementioned advantages render the centrifugal microfluidic technology even more attractive and promising for biomedical applications.
